# Explainable tabular deep learning models for antenatal cesarean delivery prediction in multiparous women

**DOI:** 10.1186/s12884-026-08934-4

**Published:** 2026-03-16

**Authors:** Emre Yalçın, Hayriye Tanyıldız, Serpil Aslan, Süleyman Cansun Demir, Mete Sucu, Fatma İşlek Uzay, Serdar Aykut, Ece Meltem Yalçın, Ayşe Biçer

**Affiliations:** 1 Department of Obstetrics and Gynecology, Division of Perinatology, Cukurova University School of Medicine, Adana, 01330 Türkiye; 2 Department of Software Engineering, Faculty of Engineering and Natural Sciences, Malatya Turgut Ozal University, Malatya, 44210 Türkiye; 3 Faculty of Medicine, Fırat University, Elazığ, 23119 Türkiye; 4 Department of Bioengineering, Faculty of Engineering and Natural Sciences, Malatya Turgut Ozal University, Malatya, 44210 Türkiye

**Keywords:** Cesarean Delivery Prediction, Multiparous Women, Tabular Deep Learning, MLP, CBAM, Deep Learning, Clinical Decision Support

## Abstract

**Background/Objectives:**

Globalincreases in cesarean section (C-section) rates, often exceeding medical necessity, highlight the need for accurate antenatal prediction to support evidence-based birth planning. Reliable prediction of delivery mode is essential for reducing maternal and neonatal morbidity, improving clinical decision-making, and optimizing resource allocation. This study analyzes a publicly available dataset of 460 multiparous women, including 18 obstetric and antenatal variables, published by Yimer and Mekonnen.

**Methods:**

Deep learning architectures were systematically evaluated for predicting delivery mode in multiparous pregnancies. Classical Multilayer Perceptrons (MLPs) served as baseline models, while modern tabular deep learning methods were assessed as advanced alternatives. Preprocessing included multiple imputation, outlier removal, and class balancing via SMOTE. Feature selection was performed using a hybrid Boruta–clinical expert strategy. Hyperparameters were tuned through Random Search. To improve interpretability, an explainability pipeline integrating SHAP and LIME was incorporated.

**Results:**

Optimized MLPs produced modest performance gains, but dedicated tabular models demonstrated clear superiority. TabNet achieved the highest performance, with an ROC-AUC of 0.79 and a PR-AUC of 0.74, attributed to its attention and masking mechanisms and robust handling of minority classes. TabPFN and CBAM-MLP yielded stable and balanced results, whereas FT-Transformer showed competitive yet comparatively moderate accuracy.

**Conclusions:**

The findings demonstrate that modern tabular deep learning approaches, particularly TabNet, surpass baseline MLP architectures in terms of accuracy, explainability, and clinical applicability for predicting C-section in multiparous women. This study presents the first comprehensive and explainable comparison of tabular deep learning models tailored to multiparous pregnancies, combining hybrid Boruta–expert feature selection with SHAP and LIME interpretability. TabNet emerges as the most promising candidate for integration into clinical decision support systems, contributing substantially to AI-driven strategies for addressing rising global C-section rates.

**Graphical Abstract:**

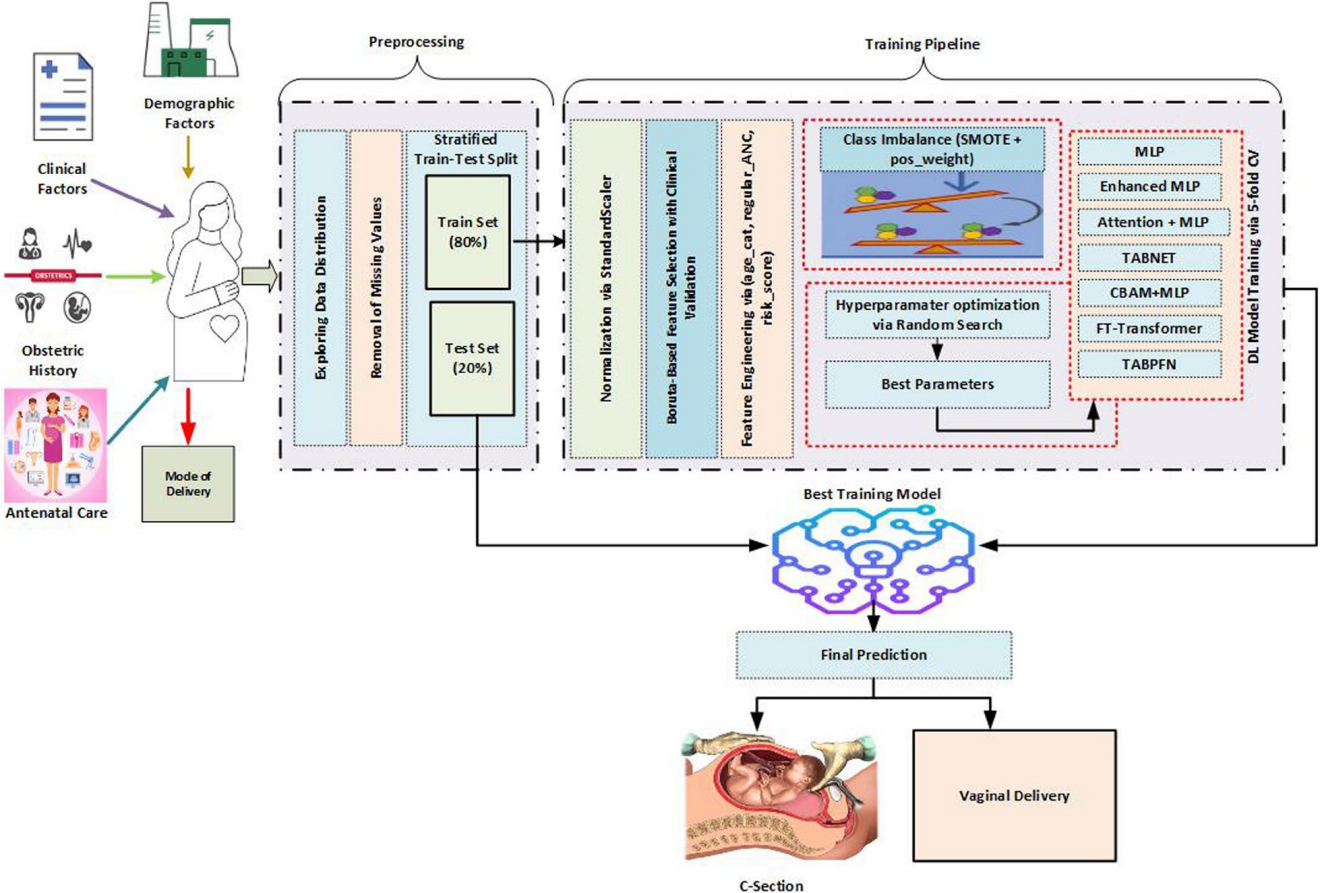

## Introduction

Cesarean section (CSE) is a frequently used surgical intervention in modern obstetric practice to protect maternal and fetal health. According to the World Health Organization, approximately 21% of births worldwide are performed by C-section, and this rate is showing an epidemiological trend, especially in developing countries [1]. The increase in the incidence of secondary C sections, especially in multiparous pregnant women with a history of primary C sections, creates a multifaceted problem that directly impacts not only clinical management but also patient safety, health policies, and the economic sustainability of healthcare services. While a C-section can be a life-saving intervention when medically required, unnecessary procedures elevate the risk of maternal complications and neonatal complications [2]. Therefore, decisions regarding the mode of delivery must be made antenatally through a careful risk-benefit analysis.

Despite the clinical importance of predicting delivery mode antenatally, existing studies do not adequately address the specific risks faced by multiparous women. Moreover, three critical methodological limitations persist in the literature: (1) most predictive models focus on nulliparous or high-risk pregnancies rather than multiparous populations; (2) prior work insufficiently integrates modern tabular deep learning methods, robust feature engineering, and explainability; and (3) comparative evaluations of attention-based architectures for clinical tabular data remain scarce.

In recent years, data-driven modeling studies for predicting the mode of birth have become a notable research area, not only for their methodological innovations but also for their contributions to the development of obstetric clinical decision support systems. Artificial intelligence and machine learning approaches, thanks to their ability to capture nonlinear patterns in multivariate data, have demonstrated superior performance compared to traditional statistical methods. Indeed, a systematic review conducted by Elhabeeb et al. [3] demonstrated the transformative potential of AI in obstetric decision support processes. Similarly, AlSaad et al. [4] reported that various AI algorithms achieved high predictive power in delivery-mode prediction. Fernández et al. [5] showed that ML-based decision support systems reached accuracy levels above 95%. Zhou [6] further emphasized the superiority of AI models over classical methods. However, reliable and generalizable models require rigorous methodological design, including appropriate handling of missing data, class imbalance, feature selection, and explainability.

Although numerous delivery-mode prediction models exist, most focus on nulliparous, high-risk, or intrapartum cases. Multiparous women, particularly those with prior cesareans, hypertensive disorders, or antepartum hemorrhage, exhibit distinct risk profiles, underscoring the need for sensitive, explainable, and clinically applicable models tailored to this population [7]. Several recent studies reflect this need: Kim et al. [8] predicted emergency cesareans in multicenter datasets; Yang et al. [9] estimated VBAC success early in pregnancy; Islam et al. [10] compared ML models on an extensive birth registry; and Wang et al. [11] developed a risk nomogram with intense discrimination. In addition, Raihen and Akter [12] demonstrated that traditional machine-learning classifiers can effectively capture maternal health risks, further highlighting the value of robust predictive modeling approaches for obstetric populations [12]. Yimer and Mekonnen [13] also highlighted the value of population-specific modeling for multiparous women in low- and middle-income countries.

Recent advances in attention-based architectures for tabular data demonstrate that self-attention mechanisms and hybrid attention–MLP designs can substantially strengthen representation learning in structured clinical datasets. Qi et al. introduced an attention-enhanced MLP supported by a privacy-preserving ACCT-GAN framework, reporting notable gains in medical tabular prediction tasks [14]. Building on this progress, Amekoe et al. showed that emerging attention-based interpretable models can achieve competitive accuracy while maintaining explanation robustness, further evidencing the maturation of attention mechanisms in tabular learning [15]. Along similar lines, Konstantinov and Utkin proposed the AFEX framework, which employs an attention-like mechanism to generate feature-specific shape functions and model pairwise interactions, offering a compelling interpretable alternative for tabular prediction tasks [16]. In parallel, Sumon et al. introduced CardioTabNet. This transformer-driven hybrid architecture captures complex feature interactions in clinical datasets. It delivers state-of-the-art predictive performance, underscoring the accelerating evolution of attention-based approaches in healthcare analytics [17]. Collectively, these studies position TabNet within a rapidly expanding ecosystem of attention-oriented models that seek to advance representation learning and interpretability in tabular domains.

The TabNet model, which demonstrates the power of deep learning in structured tabular data, was proposed by Arik et al. [18]. Thanks to its components, such as attention mechanisms, masking, and automatic feature selection, it has surpassed most traditional ML methods. Furthermore, Rahman et al. [19] reported in their study on the effect of class imbalance on model performance that KNN and Random Forest algorithms achieved the highest accuracy after employing strategies such as ROSE, SMOTE, and random oversampling. However, deep learning-based, hyperparameter-optimized, and highly explainable models developed explicitly for multiparous pregnancies remain limited in the existing literature.

Based on this deficiency, this study systematically compares different deep learning architectures for predicting cesarean delivery in multiparous pregnancies during antenatal care. The modeling process began with the basic MLP architecture and was deepened with extended versions based on Attention and CBAM. Then, modern tabular approaches, the FT-Transformer and TabPFN Classifier, were evaluated. In the final stage, the TabNet model, developed explicitly for tabular data, was applied, providing both explainability and high accuracy. During data preprocessing, outliers were identified using descriptive statistics, class imbalance was corrected using the SMOTE strategy, and no imputation was required as the dataset contained no missing values. Feature selection was optimized using the Boruta algorithm and clinical expert opinions; hyperparameters were tuned using Random Search, and the learning rate was dynamically updated using the ReduceLROnPlateau strategy.

In this regard, this study offers three key contributions to the literature: (1) the first comprehensive and explainable deep learning-based cesarean section prediction model explicitly developed for multiparous pregnancies; (2) a comparative analysis of state-of-the-art architectures operating on tabular data for obstetric prediction; and (3) the proposal of an explainable and reliable decision support system suitable for clinical integration. Furthermore, the methodological novelty of this work lies not in proposing a new variant of TabNet, but in its integrated framework that combines a hybrid feature selection strategy (Boruta-based automated selection complemented by obstetric clinical expertise) with a unified explainability pipeline leveraging SHAP, LIME, and TabNet’s inherent feature-masking mechanism. In these respects, the study not only fills a significant gap in the existing literature but also introduces a structured, clinically grounded modeling framework that enables the practical, scalable application of AI-based methods for obstetric risk stratification.

## Materials and methods

In this study, a comparative performance analysis was conducted by applying deep learning models with different architectural structures to a classification task. The evaluated models included a baseline multilayer perceptron (MLP), an enhanced MLP architecture, MLP variants enriched with attention mechanisms (Self-Attention and CBAM), and TabNet, a specifically designed architecture for tabular data. Additionally, modern tabular modeling approaches, such as the FT-Transformer and the TabPFN Classifier, were incorporated into the analysis. The architectural characteristics of each model, along with the details of the experimental setup, are presented in this section.

### Dataset and pre-processing

The dataset used in this study includes clinical, obstetric, and antenatal records of multiparous pregnant women with at least one prior birth. The data were compiled from the open-access dataset published by Yimer and Mekonnen [13]. The dataset consists of 18 variables, including the target variable mode_delivery and several predictors related to pregnancy history, maternal health conditions, and birth outcomes. Detailed descriptions of all variables are provided in Table 1.

**Table 1:** List of variables in the dataset with definitions

Variable	Definitions
participant_id	Participant ID
age	Mother’s age
gravidity	Total number of pregnancies
parity	Number of live births
abortion	Number of miscarriages
IUFD	Number of intrauterine fetal deaths
Preterm_d	Number of premature births
İnstrumental_d	Number of instrumental deliveries (vacuum, forceps, etc.)
CS	Number of cesarean deliveries
mode_delivery_last	Type of last delivery (1: vaginal, 2: cesarean? )
DM	Presence of diabetes (1: absent, 2: present)
PROM	Premature rupture of membranes
PTL	Preterm labor
APH	Antepartum hemorrhage
HDP	Hypertensive disorders of pregnancy
Mode_delivery	Type of current delivery (label - target may vary)
Medical_illness	Other medical conditions (1: absent, 2: present)
Number_ANC_cat	Number of antenatal checkups (categorical)
residence	Residence (1: rural, 2: urban)

The final dataset included 460 multiparous women, of whom 369 had vaginal deliveries (80.2%), and 91 underwent cesarean Sect. (19.8%). To enable robust model evaluation, the data were partitioned into training (80%) and test (20%) sets using stratified sampling to maintain class proportions. As the dataset contained no missing values, no imputation procedures were necessary. All subsequent preprocessing steps, including feature engineering and SMOTE-based resampling, were performed exclusively on the training set to prevent information leakage. After the initial split, the training set was further assessed using 5-fold stratified cross-validation, which served as the internal validation framework for model construction and hyperparameter optimization. The external test set remained untouched throughout the process, preserving its original distribution and providing an unbiased estimate of final model performance.

Each observation contains antenatal, obstetric, and demographic attributes. Before model development, the target variable mode_delivery was converted to a binary outcome (cesarean = 1, vaginal = 0). Due to class imbalance, a combination of data-level and algorithm-level techniques was applied. At the data level, the Synthetic Minority Over-sampling Technique (SMOTE) was used to increase the representation of cesarean cases. At the algorithm level, the pos_weight parameter was incorporated into the Binary Cross-Entropy loss function (BCEWithLogitsLoss) to enhance the model’s sensitivity to the minority class.

To strengthen predictive performance, three clinically meaningful variables were engineered based on obstetric knowledge and expert opinion:

- age_cat: Advanced maternal age indicator (≥ 35 years)
- regular_ANC: Regular antenatal care (≥ 4 visits)
- risk_score: Composite risk index (PROM, HDP, and APH)

These engineered variables were combined with existing predictors after excluding non-informative identifiers such as Unnamed: 0 and participant_id.

Feature selection was performed using the Boruta algorithm, a Random Forest–based wrapper method that identifies statistically important predictors while eliminating irrelevant or noisy features [20]. Boruta identified mode_delivery_last as the only statistically confirmed important feature. However, obstetric clinical experts emphasize that HDP, APH, PROM, and previous cesarean delivery are strong determinants of cesarean risk. Therefore, clinically critical variables that Boruta did not select were retained in the model. This hybrid approach, combining automated data-driven selection with clinical expertise, yielded a more robust and clinically meaningful feature set. The final feature set used across the modeling pipeline, therefore, included: age, mode_delivery_last, HDP, APH, PROM, medical_illness, age_cat, regular_ANC, risk_score.

Collectively, these preprocessing, feature engineering, and feature selection procedures were designed to enhance predictive accuracy, interpretability, and model robustness, ensuring suitability for practical use in obstetric decision support.

### Baseline MLP (Multilayer Perceptron)

**Fig. 1:**
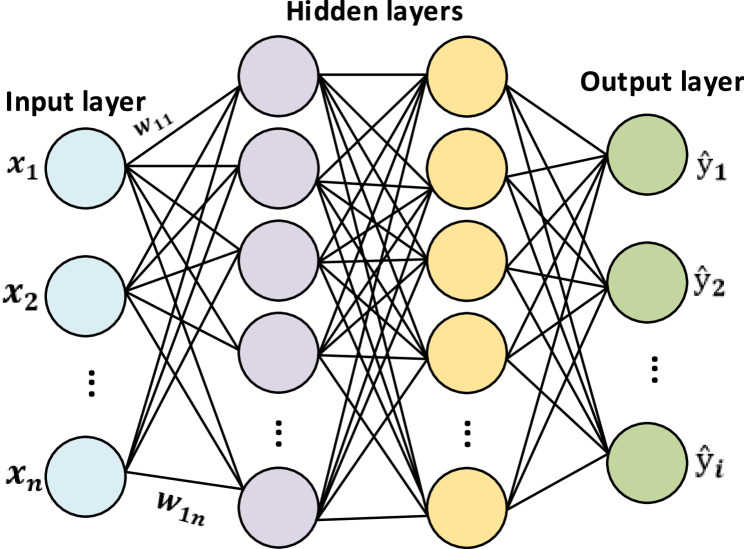
The framework of the baseline MLP architecture

The Multilayer Perceptron (MLP) [21] is one of the simplest artificial neural network architectures and is frequently adopted as a baseline model for structured data. In this study, the baseline MLP model consisted of two hidden layers with 64 and 32 neurons, respectively, following the input layer, and a sigmoid-activated output layer. To enhance the model’s ability to capture nonlinear patterns, each hidden layer employed the Rectified Linear Unit (ReLU) activation function. MLP models are particularly effective in learning complex and nonlinear data structures, enabling the development of strong feature representations and achieving reliable classification performance. The overall architecture of the baseline MLP model is illustrated in Fig. 1. In this study, the baseline MLP was utilized as the reference model against which the performance of more advanced architectures was compared.

The general mathematical formulation is expressed as:

1 $${h}_{1}=ReLU\left({W}_{1}{x}+{b}_{1}\right),$$

2 $${h}_{2}=ReLU\left({W}_{2}{h}_{1}+{b}_{2}\right)$$

3 $$\hat{\mathrm{y}} = \sigma (W_{3}h_{2} + b_{3}),$$

4 $$L_{BCE} = - \frac{1}{N} \sum [y_{i}\ \log (\hat{\mathrm{y}}_{i}) + (1 - {y}_{i}) \log (1 - \hat{\mathrm{y}}_{i})]$$

where 𝑥 denotes the input feature vector, ${W}_{1}$, ${W}_{2}$, ${W}_{3}$ and ${b}_{1}$, ${b}_{2}$, ${b}_{3}$​ represent the weight matrices and bias terms of each layer, respectively. ${h}_{1}$ and ${h}_{2}$ denote the hidden layer outputs, 𝜎 ( ⋅ ) is the sigmoid activation function, and $ŷ$ yrepresents the model’s final prediction. The loss function ${L}_{BCE}$ ​ corresponds to the Binary Cross-Entropy, which evaluates the discrepancy between predicted probabilities and the true class labels ${y}_{i}$ .

### Enhanced MLP

**Fig. 2:**
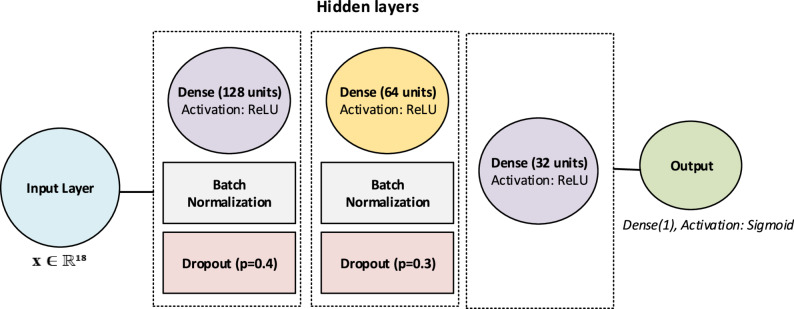
The framework of the enhanced MLP architecture

To enhance the depth and generalizability of the baseline MLP, several regularization techniques proposed in the literature were incorporated into the architecture [22]. The improved model comprises three hidden layers with 128, 64, and 32 neurons, respectively, and integrates Batch Normalization (BN) and Dropout layers. BN layers are applied after each hidden layer to stabilize the learning dynamics, while Dropout prevents overfitting by randomly deactivating neurons during training. Additionally, the learning rate is dynamically adjusted using the ReduceLROnPlateau strategy, which contributes to more efficient convergence. Compared to the classical MLP, this design enables the model to capture more complex patterns and achieve a higher representational capacity [23]. The framework of the enhanced MLP architecture is shown in Fig. 2.

The general mathematical formulation is expressed as:

5 $$BN\left(z\right)=\frac{\gamma\left(z-{\mu}_{B}\right)}{\sqrt{{\sigma}_{B}^{2}+\epsilon}}+\beta,$$

6 $$Dropout\left(h\right)=h\odot m,{m}_{j}\sim Bernoulli\left(p\right).$$

where 𝑧 denotes the input to the batch normalization layer,${\mu}_{B}$ and ${\sigma}_{B}^{2}$ are the batch-wise mean and variance, respectively, and 𝛾 and 𝛽 are learnable scaling and shifting parameters. In Eq. (6), ℎ refers to the hidden activations, 𝑚 is the dropout mask sampled from a Bernoulli distribution with probability 𝑝, and ⊙ denotes element-wise multiplication.

### Attention + MLP

**Fig. 3:**
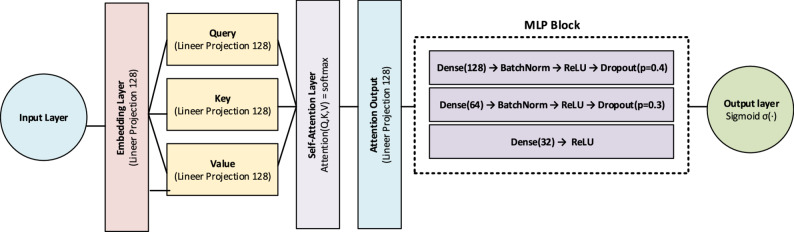
The framework of the Attention + MLP architecture

The self-attention mechanism, popularized through Transformer-based architectures, enables the modeling of contextual dependencies among input features [24]. In this study, a self-attention layer was applied to the input features, allowing each feature to learn its interactions with the others before being passed to the conventional MLP layers. This approach enhances classification performance, particularly in tabular datasets where inter-feature dependencies are strong. By incorporating self-attention, the model gains contextual awareness and reveals hidden patterns within the data [25]. The framework of the Attention + MLP architecture is shown in Fig. 3.

The general mathematical formulation is expressed as:

7 $$Q={W}_{Q}x,K={W}_{K}x,V={W}_{V}x$$

8 $$Attention\left(Q,K,V\right)=softmax\left(\frac{Q{K}^{T}}{\sqrt{{d}_{k}}}\right)V$$

where 𝑥 represents the input feature vector, $${W}_{Q}$$,​ $${W}_{K}$$$${W}_{V}$$, are the projection weight matrices for the query, key, and value vectors, respectively. 𝑄, 𝐾, 𝑉 denote the transformed query, key, and value matrices, while $${d}_{k}$$​ is the dimension of the key vectors used for scaling. The softmax function ensures that attention weights form a probability distribution across the features.

### CBAM + MLP

**Fig. 4:**
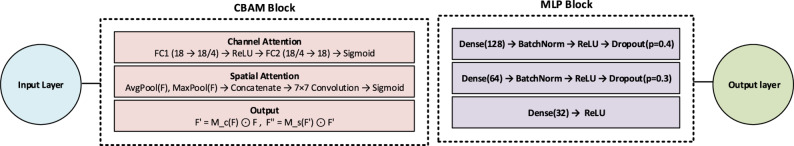
The framework of the CBAM + MLP architecture

The Convolutional Block Attention Module (CBAM) is an attention-based mechanism originally designed for computer vision tasks [26]. In this study, the CBAM block was adapted for tabular data to apply both channel attention and spatial attention on the input features. The channel attention mechanism learns which features are more informative, while the spatial attention emphasizes the most relevant parts of the input samples. The CBAM outputs are subsequently passed through conventional MLP layers to perform classification. This structure allows the model to focus not only on all features equally but also on the most significant ones, thereby improving interpretability and predictive performance. Previous studies have demonstrated that CBAM enhances feature-selective learning and contributes to model explainability [27]. The framework of the CBAM + MLP architecture is shown in Fig. 4.

The general mathematical formulation is expressed as:

9 $${M}_{c\left(F\right)}=\sigma\left({W}_{1}.ReLU\left({W}_{0}{F}_{avg}\right)+{W}_{1}ReLU\left({W}_{0}{F}_{max}\right)\right),$$

10 $${M}_{s\left(F\right)}=\sigma\left({f}^{7x7}\left(\left[{F}_{\left\{avg\right\}};{F}_{\left\{max\right\}}\right]\right)\right).$$

where ${F}_{avg}$ and ${F}_{max}$ ​ denote the average-pooled and max-pooled feature descriptors, respectively; ${W}_{0}$ and ${W}_{1}$ are learnable weight matrices; ReLU(⋅) represents the activation function; ${f}^{7x7}$ (⋅) indicates a convolution operation with a 7 × 7 kernel; and σ(⋅) is the sigmoid activation function that normalizes the attention maps. ${M}_{c\left(F\right)}$ and ${M}_{s\left(F\right)}$ represent the channel attention and spatial attention maps, respectively.

### TabNet

**Fig. 5:**
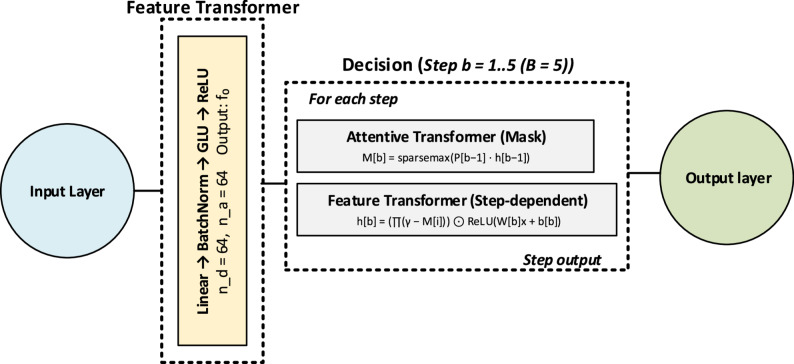
The framework of TabNet architecture

TabNet is a modern deep learning architecture specifically designed for tabular data. Unlike conventional multilayer perceptrons (MLPs), TabNet integrates feature selection, attention mechanisms, and masked learning within its structure [18]. At each decision step, the attention mechanism identifies the most informative features, and masked learning ensures that the model focuses on these selected features while processing information. This approach enables TabNet to learn more targeted and interpretable representations, rather than relying on randomly learned weights as in classical MLPs. Previous studies have demonstrated that TabNet achieves competitive or superior performance compared to traditional machine learning methods across classification and regression tasks [28]. The framework of the TabNEt architecture is shown in Fig. 5.

The general mathematical formulation is expressed as:

11 $$M\left[b\right]=sparsemax\left({P}_{b-1}{h}_{b-1}\right),$$

12 $$h\left[b\right]=\left({\prod}_{i=1}^{b-1}(\gamma-M\left[i\right])\right)\cdot ReLU\left.\left(W\left[b\right]x+b\left[b\right]\right.\right)$$

13 $$\widehat{y}=\sum\nolimits_{b=1}^{B}{\phi}_{b}\left(h\right[b\left]\right).$$

where 𝑥 denotes the input feature vector, $W\left[b\right]$ and $b\left[b\right]$ are the weight matrix and bias term at the b-th decision step, respectively. $h\left[b\right]$ represents the hidden representation at step 𝑏, while $M\left[b\right]$ denotes the feature mask generated using the sparsemax function. 𝛾 is the attenuation factor that controls the reuse of previously selected features.${\prod}_{i=1}^{b-1}(.)$ indicates the multiplicative effect of masks from previous steps, ensuring progressive feature selection. ${\phi}_{b}$ is the prediction function of the b-th decision step, and $\widehat{y}$ ​ is the final model output.

### FT-Transformer

**Fig. 6:**
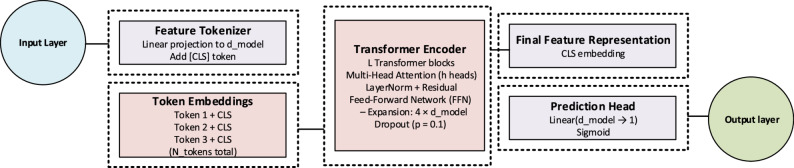
The framework of FT- Transformer architecture

The FT-Transformer (Feature Tokenizer + Transformer) introduces a Transformer-based architecture specifically tailored for tabular data and is particularly effective in modeling complex feature interactions [29]. The model utilizes a Feature Tokenizer module to convert input features into tokens, which are then processed by Transformer blocks to capture inter-feature dependencies. The multi-head self-attention mechanism enables the model to contextualize each feature with respect to the others, thereby improving representational capacity. Finally, a classification head produces the output prediction. The framework of the FT-Transformer architecture is shown in Fig. 6.

The general mathematical formulation is expressed as:

14 $$Z=Transformer\left({X}_{token}\right),$$

15 $$\widehat{y}=\sigma\left({W}_{out}Z+{b}_{out}\right).$$

where ${X}_{token}$ ​ denotes the tokenized input features, 𝑍 is the contextualized representation obtained from the Transformer,${W}_{out}$​ and ${b}_{out}$ ​ are the output layer’s weight matrix and bias term, respectively, and σ(⋅) is the sigmoid activation function that maps the output to a probability.

### TABPFN

**Fig. 7:**
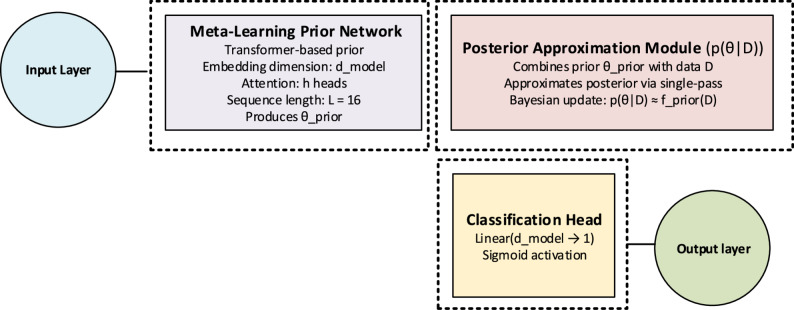
The framework of TABPFN architecture

The TabPFN Classifier (Tabular Prior-Data Fitted Network) is a modern deep learning architecture that leverages a pretrained Bayesian prior distribution to enable fast and accurate predictions on tabular datasets [30]. By adopting a “Prior-Data Fitted Network” approach, TabPFN performs meta-learning across multiple data subspaces during training, which allows the model to achieve high accuracy even with limited sample sizes. The framework of the TABPFN architecture is shown in Fig. 7.

The general mathematical formulation is expressed as:

16 $$p\left(\theta\right|D)=\frac{p\left(D \right|\theta\left)p\right(\theta\left)\right]}{p\left(D\right)}.$$

where 𝐷 denotes the training dataset and 𝜃 represents the model parameters. The TabPFN Classifier approximates this posterior distribution and applies it directly to classification tasks.

### Hyperparameter optimization

Hyperparameter optimization is the process of systematically tuning parameters that control the model architecture and training dynamics (e.g., learning rate, layer size, and regularization coefficients) in order to maximize predictive performance. In this study, hyperparameter optimization was performed using the Random Search algorithm. Compared with Grid Search, Random Search provides the advantage of exploring a broader hyperparameter space more efficiently [31].

The general mathematical formulation is expressed as:

17 $${\theta}^{*}=argmi{n}_{\theta\in{\Theta}}\mathcal{}\mathcal{L}\left({f}_{\theta}\left(X\right),Y\right).$$

where 𝜃 denotes the set of hyperparameters Θ is the hyperparameter search space, ${f}_{\theta}\left(X\right)$ represents the model predictions parameterized by 𝜃, 𝑌corresponds to the ground-truth labels, and $\mathcal{L}\left(.\right)$ is the loss function minimized during training.

### The proposed approach

In this study, we propose an explainable TabNet-based deep learning model for the antenatal prediction of cesarean delivery in multiparous women. TabNet is a modern architecture designed explicitly for tabular data, which combines attention mechanisms and feature-masking strategies to enable automatic feature selection and enhance interpretability. By focusing on the most informative features at each decision step, TabNet provides both high classification accuracy and transparent decision-making, positioning itself beyond traditional machine learning methods. Although the model architecture itself is not modified, the methodological novelty of the proposed framework arises from its task-specific adaptation to multiparous cesarean prediction and the integration of several components that extend beyond standard TabNet implementations. In particular, the approach introduces a hybrid feature-selection strategy, a multi-level explainability pipeline, and a comparative evaluation setting that situates TabNet within a broader family of tabular deep learning architectures.

The proposed approach directly addresses several challenges commonly encountered in clinical datasets, including class imbalance, complex feature interactions, and the need for interpretability in decision support. To ensure methodological rigor and domain relevance, the design incorporates innovations tailored to the characteristics of obstetric data. In the model development process, multiple methodological enhancements were incorporated:

- SMOTE-based resampling to mitigate class imbalance,
- sensitive loss functions tailored for minority class prediction,
- hyperparameter optimization via Random Search to achieve robust parameter configurations, and.
- early stopping strategies to prevent overfitting and improve generalizability.

Furthermore, the framework uniquely combines automated feature selection using the Boruta algorithm with obstetric clinical expertise, ensuring that statistically important variables and clinically essential predictors are jointly represented in the model. In addition, the explainability layer extends beyond TabNet’s intrinsic masks by integrating SHAP for global feature attribution and LIME for local interpretability, resulting in a unified and clinically coherent explanation pipeline that is not present in conventional TabNet applications.

In addition, to prevent data leakage, all preprocessing and resampling procedures (including Boruta-based feature selection, feature engineering, and SMOTE) were applied strictly to the training data after the stratified train–test split. The external test set was kept completely untouched and retained its original class distribution throughout the entire modeling process.

**Fig. 8:**
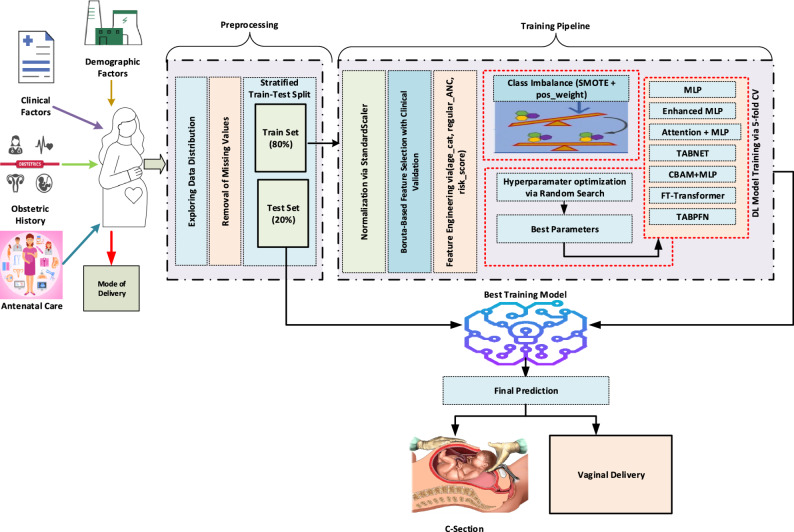
The framework of the proposed approach

This design ensures not only predictive accuracy but also reliability, transparency, and clinical integration potential. Overall, the proposed TabNet-based system should be viewed not as a direct reproduction of the original architecture but as a customized, clinically grounded decision-support framework tailored specifically to multiparous pregnancies. The overall workflow of the proposed approach, encompassing data preprocessing, model training, and evaluation, is schematically illustrated in Fig. 8.

## Results

This study assessed the performance of both classical MLP-based architectures and modern deep learning approaches designed explicitly for tabular data in predicting cesarean delivery among multiparous women. The MLP and its variants (Enhanced MLP, Attention-MLP, CBAM-MLP) were employed as baseline models to enable an objective benchmarking against state-of-the-art tabular architectures, namely TabNet, FT-Transformer, and TabPFN. To the best of our knowledge, this work is among the few studies to systematically compare traditional neural network models with advanced tabular frameworks in the context of delivery mode prediction.

Model performance was primarily evaluated using the area under the ROC (Receiver Operating Characteristic) curve and the PR (Precision–Recall) curve. While ROC curves provide a global measure of the models’ discriminative ability, PR curves are critical in this study due to the class imbalance between cesarean and vaginal deliveries, where accurate prediction of the minority class (cesarean) is critical for patient safety. Each model was assessed under both default configurations and optimized hyperparameter settings, thereby highlighting not only the influence of architectural design but also the role of hyperparameter tuning in shaping predictive performance. This dual evaluation framework provides an objective assessment of the potential of each architecture for tabular clinical data.

**Fig. 9:**
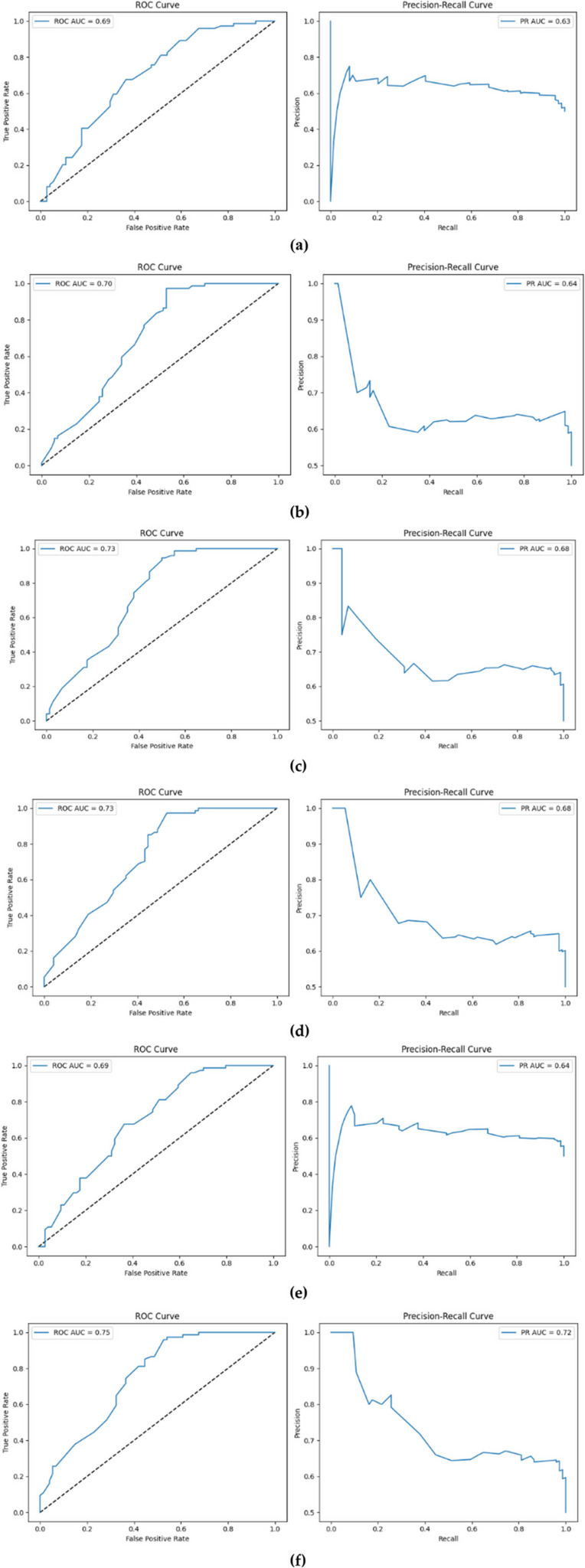
Comparison of classification performance of different deep learning architectures in predicting cesarean delivery: **a** Baseline MLP, **b** Enhanced MLP, **c** Attention + MLP, **d** CBAM + MLP, **e** FT-Transformer, **f** TabPFN

Figure 9 presents a comparative analysis of the classification performance of various deep learning architectures for predicting cesarean deliveries, evaluated through Receiver Operating Characteristic (ROC) and Precision–Recall (PR) curves. These metrics are crucial for evaluating both the overall discriminative capacity of the models and their ability to identify the minority class, specifically cesarean deliveries, accurately. The baseline MLP model (Fig. 9a) exhibited limited discriminative power, with an ROC-AUC of 0.70 and a PR-AUC of 0.62. The relatively low PR-AUC highlights the model’s insufficiency in predicting the positive class (cesarean) under conditions of class imbalance. The enhanced MLP (Fig. 9b), despite incorporating Batch Normalization, Dropout, and dynamic learning rate adjustments, achieved only marginal improvements, with an ROC-AUC of 0.70 and a PR-AUC of 0.64. The Attention + MLP model (Fig. 9c) yielded an ROC-AUC of 0.72 and a PR-AUC of 0.65, indicating that attention mechanisms contributed to capturing inter-feature relationships. However, fluctuations in the PR curve suggested instability in maintaining a balanced trade-off between precision and recall. The highest performance was observed with the CBAM-integrated MLP (Fig. 9d). Achieving an ROC-AUC of 0.77 and a PR-AUC of 0.73, this model highlights the effectiveness of combining channel and spatial attention mechanisms to generate more selective and meaningful feature representations. The ROC curve’s distinct separation from the random classification line indicated the model’s capacity to achieve simultaneously high accurate positive rates and low false positive rates. Meanwhile, the balanced structure of the PR curve confirmed consistent and reliable performance in terms of both precision and recall.

**Fig. 10:**
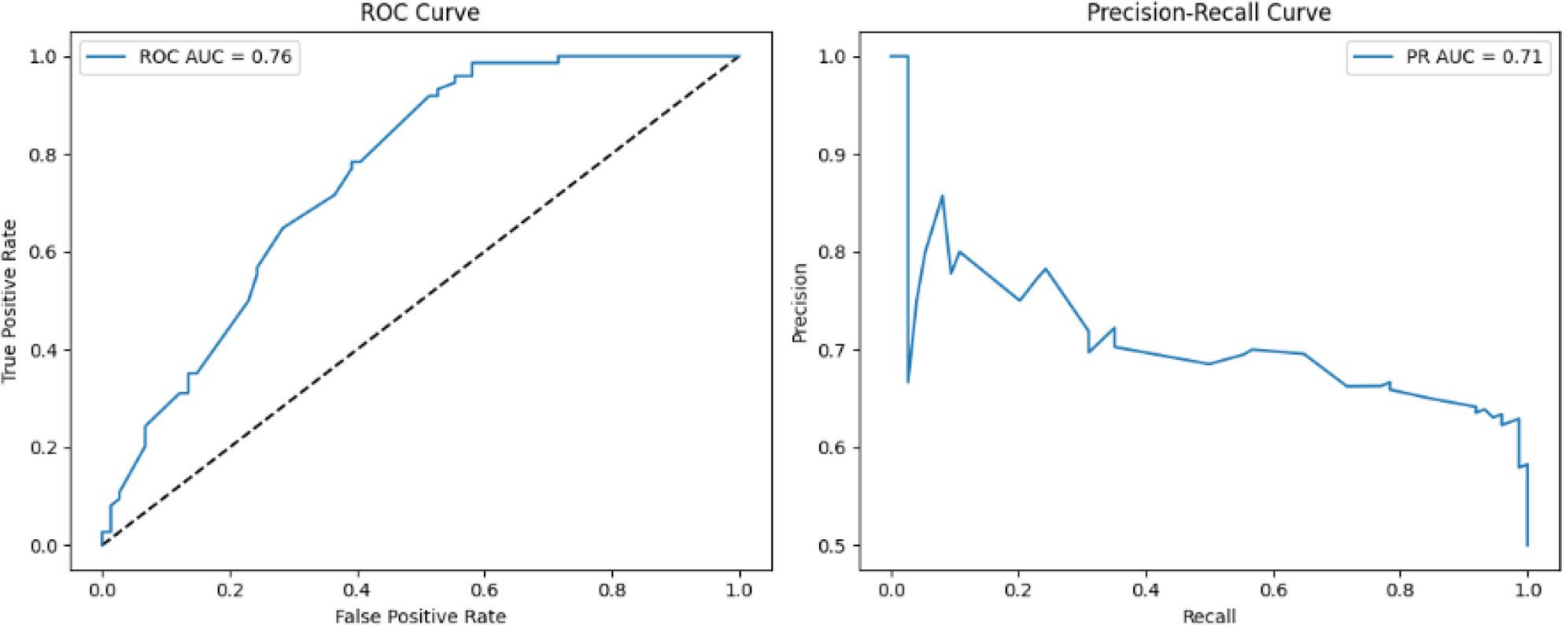
Classification performance of the TabNet model for predicting cesarean delivery

Figure 10 illustrates the classification performance of the TabNet model for predicting cesarean delivery, evaluated through Receiver Operating Characteristic (ROC) and Precision–Recall (PR) curves. The ROC-AUC value of 0.76 indicates that the model was able to effectively discriminate between positive and negative classes. The PR-AUC, calculated as 0.71, further demonstrates that the model achieved high sensitivity and acceptable precision in predicting the minority class. Although fluctuations were observed in the PR curve at lower recall levels, suggesting some performance variability at specific thresholds, the overall trend confirms the model’s reliable identification of the positive class. These results highlight the effectiveness of TabNet’s attention-based structure in feature selection and demonstrate that, compared to previous architectures, it provides a more stable, explainable, and clinically applicable performance.

### Impact of hyperparameter optimization

Properly configuring hyperparameters plays a decisive role in the overall accuracy, generalization ability, and learning dynamics of machine learning and deep learning models. In this study, various key hyperparameters, including the learning rate, number of layers, number of neurons, and dropout rate, were systematically optimized for each model architecture. This optimization process improved the classification performance of the models while also reducing the risk of overfitting. Different hyperparameter combinations were tested for each classification model used in the experimental comparisons, and the configurations that provided the highest performance were selected. The configurations containing the optimal parameter values ​​are presented in Table 2.

**Table 2:** Optimal hyperparameter configurations for the classification models following optimization

	Parameters	Value
MLP	hidden1, hidden2, dropout, lr	256, 64, 0.005, 0.2
Enhanced MLP	hidden1, hidden2, dropout, lr	512, 128, 7, 0.01
Attention-MLP	hidden1, hidden2, dropout, lr	128, 128, 0.2, 0.02
CBAM	hidden1, hidden2, dropout, lr	256, 128, 0.4, 0.005
TabNET	n_d, n_a, n_steps, gamma, lambda_sparse, lr	64, 128, 7, 1.5, 1e-05, 0.02
Ft-Transformer	ffn_dropout, lr	0.3, 0.005
TABPFN	training_steps, batch_size, optimizer	16, 32, AdamW

**Fig. 11:**
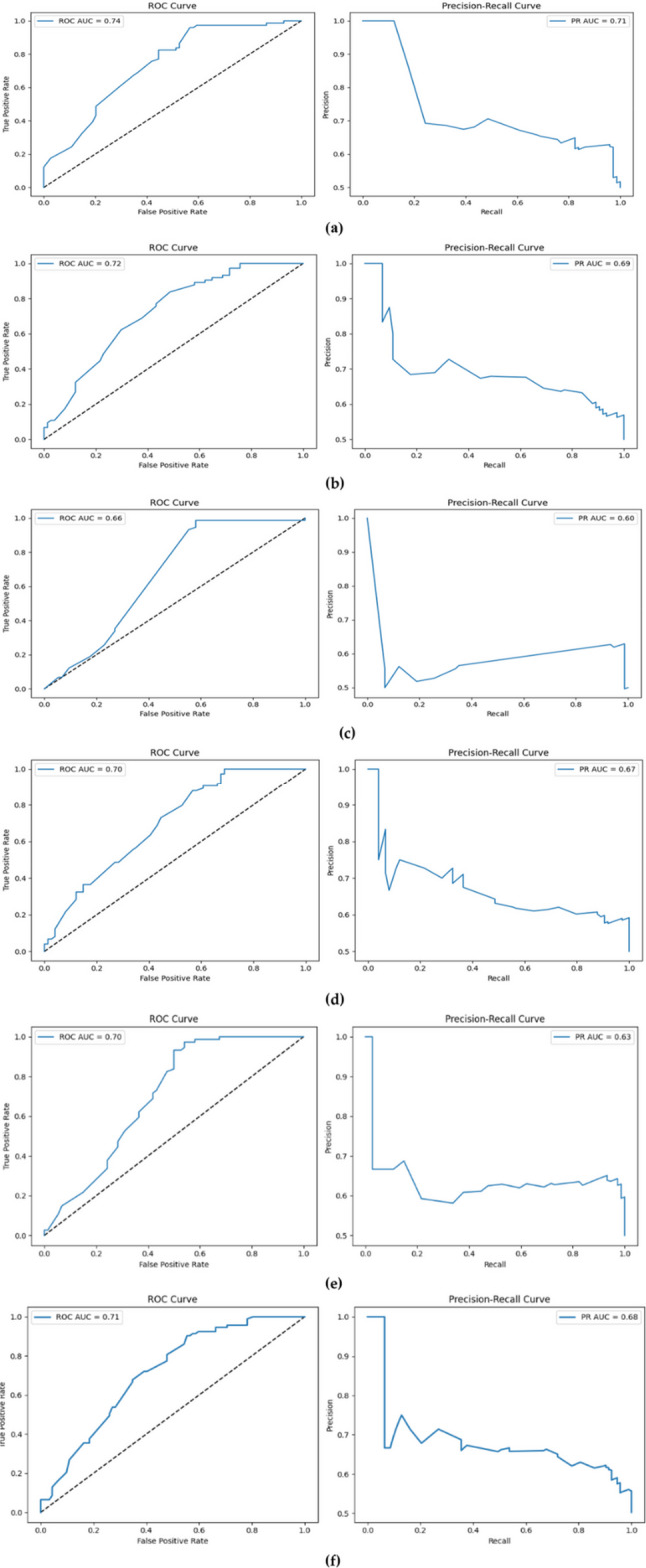
Comparison of the classification performance of different deep learning architectures after hyperparameter optimization for predicting cesarean delivery: **a** Baseline MLP, **b** Enhanced MLP, **c** Attention + MLP, **d** CBAM + MLP, **e** FT-Transformer, **f** TabPFN

Figure 11 presents the classification performance of the optimized models after hyperparameter tuning, evaluated through Receiver Operating Characteristic (ROC) and Precision–Recall (PR) curves. These metrics provide complementary insights into the models’ ability to distinguish between classes and reliably identify the minority class (cesarean deliveries). The baseline MLP model (Fig. 11a) achieved the highest accuracy and balance, with an ROC-AUC of 0.74 and a PR-AUC of 0.71. The ROC curve’s proximity to the top-left corner indicates reliable discrimination between positive and negative classes. The PR curve exhibits high precision at low recall levels; however, precision drops markedly as recall increases, indicating that while the model effectively identifies a limited number of positive cases, its performance deteriorates when attempting to capture a broader range of the positive class. The enhanced MLP model (Fig. 11b) yielded an ROC-AUC of 0.72 and a PR-AUC of 0.69, demonstrating performance comparable to the baseline. Although the deeper architecture improved pattern recognition capacity, only marginal gains in discriminative power were observed. The PR curve reveals high precision at low recall levels. However, the balance could not be maintained as recall increased, highlighting the model’s limited ability to generalize across the positive class. The Attention-MLP model (Fig. 11c) yielded an ROC-AUC of 0.66 and a PR-AUC of 0.60, indicating that the attention mechanism did not sufficiently enhance class separation for this dataset. The PR curve shows that meaningful precision was achieved only at low recall levels, with performance dropping sharply as generalization increased. These results suggest that this approach has limited clinical reliability. The CBAM-MLP model (Fig. 11d) exhibited a more balanced performance, with an ROC-AUC of 0.70 and a PR-AUC of 0.67. The PR curve indicates that precision was relatively well maintained as recall increased, suggesting that the integration of channel and spatial attention improved the consistent identification of the positive class. This highlights the model’s greater stability when applied to imbalanced datasets. Overall, while the baseline MLP achieved the highest accuracy values, the CBAM-MLP offered a more balanced and consistent learning process. The effectiveness of attention-based mechanisms varied depending on the data structure, underscoring the importance of considering explainability and generalization ability in model selection.

**Fig. 12:**
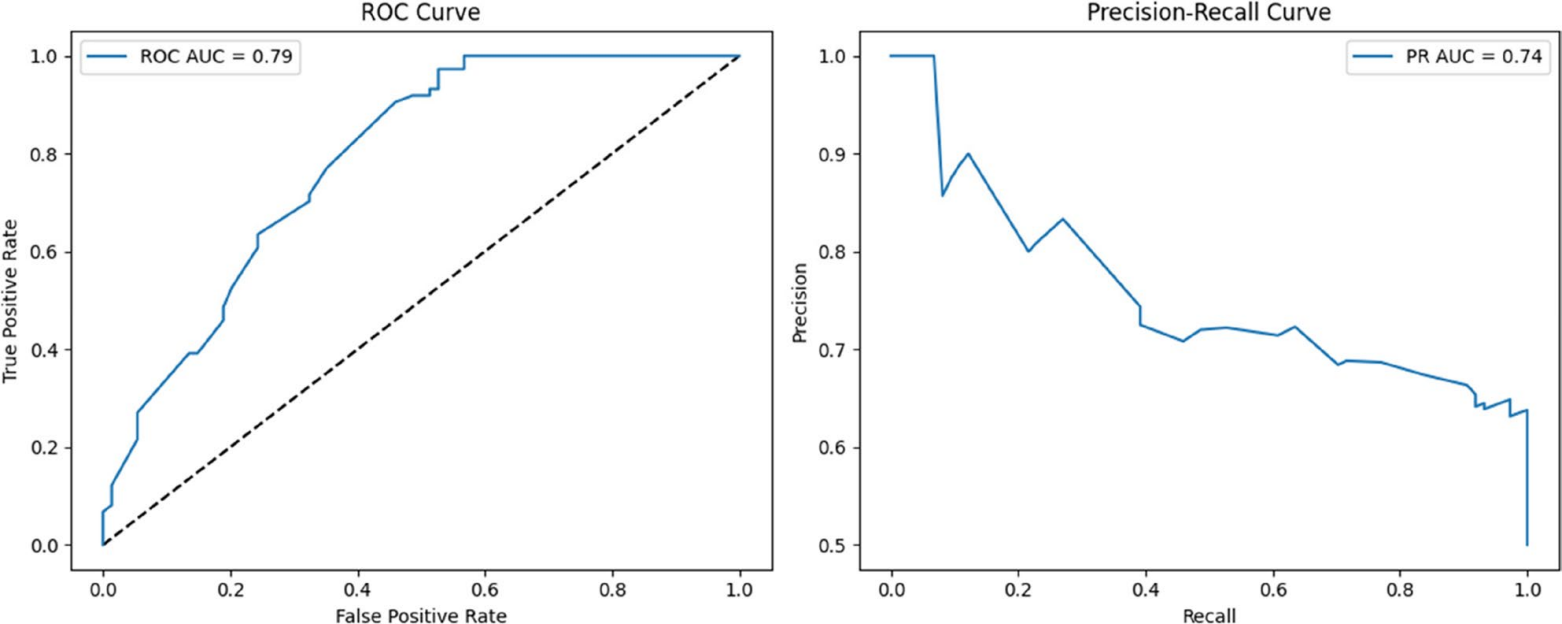
Comparison of the classification performance of TabNet after hyperparameter optimization for predicting cesarean delivery

Figure 12 illustrates the classification performance of the proposed TabNet model, evaluated through Receiver Operating Characteristic (ROC) and Precision–Recall (PR) curves. The ROC-AUC was calculated as 0.79, indicating that the model effectively discriminated between positive and negative classes with high accuracy. The ROC curve’s proximity to the upper-left corner further demonstrates the model’s ability to simultaneously maintain a high true positive rate and a low false positive rate. The area under the PR curve (PR-AUC) was 0.74, suggesting that the model was capable of identifying positive cases with both high precision and high recall. An examination of the PR curve shows that while precision remained high at lower recall levels, it gradually decreased as recall increased.

Nevertheless, the overall curve structure supports the conclusion that TabNet provides a balanced, sensitive, and generalizable classification performance for the minority class. When compared with alternative architectures, TabNet outperformed previous models in terms of both ROC-AUC and PR-AUC metrics. Although the baseline and enhanced MLP architectures achieved competitive accuracy, their limited explainability and performance degradation in covering a broader range of positive cases represent notable limitations in clinical contexts. Similarly, attention-based architectures, such as Attention-MLP and CBAM-MLP, provided marginal improvements in sensitivity under specific conditions, but their relatively low accuracy hindered overall classification reliability. Taken together, these findings highlight TabNet not only as the numerically best-performing model but also as the most balanced in terms of explainability and generalization capacity, underscoring its clinical applicability for predicting cesarean delivery.

To determine whether the performance differences between models were statistically significant, pairwise ROC-AUC comparisons were conducted using the non-parametric DeLong test, with TabNet serving as the refer-ence model. The analysis showed that TabNet significantly outperformed all other architectures (*p* < 0.05). The most significant differences were observed against attention-based models such as Attention-MLP and CBAM-MLP (p < 0.001 and p = 0.006, respectively). Classical MLP variants and other tabular architectures (FT-Transformer and TabPFN) also exhibited statistically lower ROC-AUC values compared with TabNet. These findings confirm that TabNet's superiority is not only numerical but also statistically significant. The detailed sta-tistical results are presented in Table 3.

**Table 3:** DeLong statistical comparison of ROC-AUC scores across models (reference: TabNet)

Comparison	AUC Difference	*p*-value	Interpretation
TabNet vs. MLP	0.05	0.041	Significant
TabNet vs. Enhanced MLP	0.0687	0.022	Significant
TabNet vs. Attention-MLP	0.1272	< 0.001	Highly significant
TabNet vs. CBAM-MLP	0.09	0.006	Highly significant
TabNet vs. FT-Transformer	0.09	0.008	Highly significant
TabNet vs. TabPFN	0.08	0.014	Significant

Table 4 presents a comparison of the ROC-AUC and PR-AUC scores for the default and hyperparameter-optimized versions of the deep learning architectures evaluated in this study. The findings indicate that hyperparameter optimization provided significant performance improvements for some models, while having limited or adverse effects for others. Specifically, the TabNet model demonstrated the highest overall performance among all models, improving its ROC-AUC from 0.7569 to 0.79 and its PR-AUC from 0.7054 to 0.74 upon optimization. This result confirms that TabNet’s attention and masking mechanisms, specifically designed for tabular data, enable a more selective and explainable learning process when hyperparameter settings are optimized. Similarly, the Advanced MLP model showed a significant increase in positive class recognition, increasing the ROC-AUC value from 0.7018 to 0.7213 and the PR-AUC value from 0.6418 to 0.6864 after optimization. However, it is also observed that hyperparameter optimization does not always have a positive effect. In the Attention-MLP model, the ROC-AUC decreased from 0.72 to 0.66, and the PR-AUC from 0.69 to 0.60 after optimization. Similarly, in the CBAM-based model, the ROC-AUC decreased from 0.77 to 0.6976, and the PR-AUC decreased from 0.73 to 0.6656. These findings suggest that excessive parameterization, especially in architectures that incorporate attention mechanisms, can increase the risk of overfitting; therefore, hyperparameter tuning should be more sensitive. Other modern approaches developed for tabular data are also noteworthy. FT-Transformer achieved similar results before and after optimization (ROC-AUC ≈ 0.69–0.70; PR-AUC ≈ 0.63), demonstrating that this architecture offers relatively stable but limited performance. TabPFN, on the other hand, despite working with pre-trained parameters, saw a drop in ROC-AUC from 0.75 to 0.71 and a drop in PR-AUC from 0.71 to 0.68. This result suggests that while TabPFN offers a strong starting point, it is highly sensitive to the dataset and problem context. In conclusion, while hyperparameter optimization has the potential to improve model performance, this process is not equally effective for all architectures. Models specifically designed for tabular data, such as TabNet and TabPFN, offer robust and explainable solutions for predicting cesarean delivery in multiparous women when configured correctly. Therefore, it is crucial to adjust the hyperparameters in a model-specific manner, considering the unique structural features of each architecture.

**Table 4:** Comparative performance metrics of the evaluated deep learning models

Model	Optimized		Default	
	ROC AUC	PR AUC	ROC AUC	PR AUC
MLP	0.74	0.71	0.7018	0.6239
Enhanced MLP	0.7213	0.6864	0.7018	0.6418
Attention + MLP	0.6628	0.5963	0.7218	0.6471
TABNET	0.79	0.74	0.7569	0.7054
CBAM + MLP	0.70	0.67	0.73	0.68
FT-Transformer	0.70	0.63	0.69	0.63
TABPFN	0.71	0.68	0.75	0.71

### Ablation analysis on feature selection

An ablation analysis was performed to quantify the contribution of automated and clinically engineered features to TabNet’s predictive performance. The Boruta algorithm identified mode_delivery_last as the sole statistically significant feature, while three clinically relevant variables (age_cat, risk_score, regular_ANC) were retained based on obstetric expertise, yielding a nine-feature model. As shown in Table 5, removing the Boruta-selected feature (M0) or excluding clinically engineered variables (M1) resulted in measurable declines in ROC-AUC and PR-AUC. The full pipeline (M2), which integrates Boruta selection with clinical feature engineering, achieved the highest performance, demonstrating that automated selection and domain knowledge provide complementary gains in discrimination and minority-class detection.

**Table 5:** Performance impact of feature selection and clinically engineered variables in the TabNet model

Model	Description	ROC-AUC	PR-AUC
M0	Without Boruta-selected feature	0.603	0.624
M1	Without clinically engineered features	0.618	0.631
M2	Full pipeline (Boruta + engineered features)	0.754	0.702

These findings confirm that both automated feature selection and clinical expertise contribute meaningfully to improved discrimination and minority-class detection in cesarean prediction.

### Ablation analysis on class imbalance handling

Effective handling of class imbalance is critical for minority-class prediction; therefore, three configurations were compared to assess the contribution of each strategy. The evaluation considered models with (1) no SMOTE and no class weighting, (2) SMOTE only, and (3) the combined approach used in the final model (SMOTE + pos_weight), using PR-AUC as the imbalance-sensitive metric. As shown in Table 6, PR-AUC increased progressively across the configurations: SMOTE alone provided moderate improvement, whereas the combined strategy achieved the highest performance (0.74). These findings indicate that data-level oversampling and algorithm-level weighting operate synergistically, yielding the most stable and clinically meaningful minority-class predictions.

**Table 6:** Impact of imbalance-handling strategies on PR-AUC performance

Model Condition	PR-AUC
No SMOTE, No Class Weight	0.61
SMOTE Only	0.66
SMOTE + pos_weight (Final)	0.74

### Explainability analysis

In the absence of a universally accepted quantitative explainability metric for tabular medical AI models, SHAP contribution scores and LIME-based local explanations are widely regarded in the clinical AI literature as practical and reliable interpretability measures. Therefore, this study evaluates explainability using both global and local feature-attribution analyses and validates their consistency across multiple XAI methods. To enhance the transparency and clinical interpretability of the proposed TabNet-based decision support system, comprehensive explainable artificial intelligence (XAI) techniques were applied. Both global and instance-level explanations were generated using SHAP (SHapley Additive exPlanations) and validated through LIME-based consistency checks. These analyses provide insight into how individual features contributed to the model’s predictions and demonstrate that the decision process aligns with well-established clinical risk factors.

#### SHAP-based global and local interpretations

**Fig. 13:**
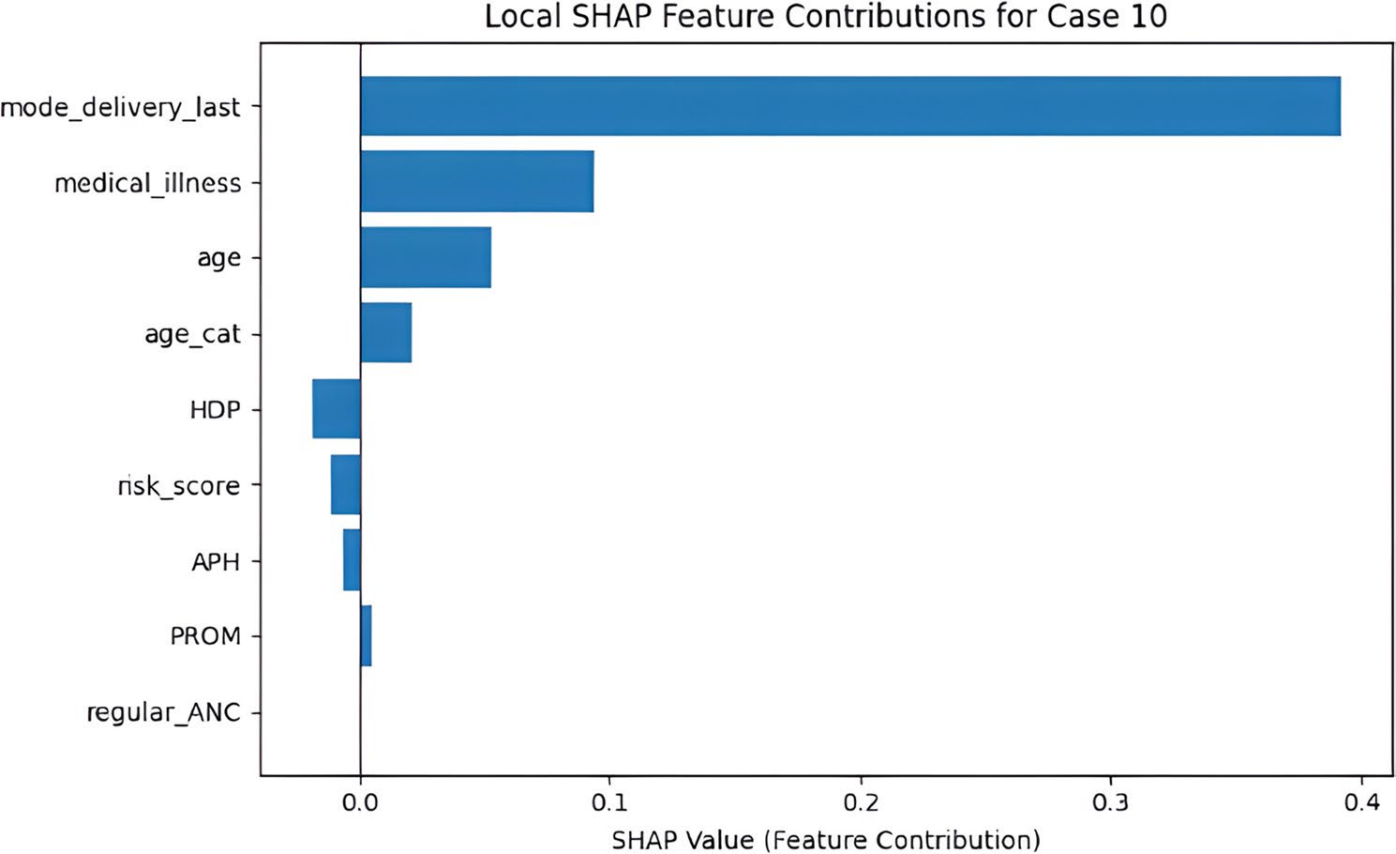
Local SHAP feature contribution plot for the selected test case

Global SHAP analysis identified the dominant predictors influencing cesarean delivery decisions across the entire dataset. The most influential features included mode_delivery_last, medical_illness, age, age_cat, and HDP, all of which are known clinical determinants of delivery mode. These high-impact variables indicate that the model systematically prioritizes obstetric history, maternal comorbidity, and maternal age in its decision-making process. To further investigate the model’s reasoning at the individual level, a representative case from the test set was examined using local SHAP values. In this case, the model predicted a high probability of cesarean delivery. The SHAP values of the features contributing to this prediction are presented in Table 7, and the corresponding SHAP feature contribution plot is shown in Fig. 13.

**Table 7:** SHAP-based local feature contributions for the selected test case

Feature	Value	SHAP Contribution
mode_delivery_last	-0.480	+ 0.3919
medical_illness	0.314	+ 0.0942
age	-0.947	+ 0.0531
age_cat	-0.430	+ 0.0282
HDP	0.308	−0.0191
risk_score	0.405	−0.0112
APH	0.235	−0.0065
PROM	0.182	−0.0049
regular_ANC	0.000	0.0000

As shown in Table 7, mode_delivery_last emerged as the strongest positive contributor (+ 0.3919), indicating that a history of cesarean delivery exerted the greatest influence on the model’s prediction. This finding is clinically expected, as obstetric history is one of the most decisive determinants of delivery mode. The second most influential variable, medical_illness (+ 0.0942), aligns with established evidence linking maternal comorbidities to elevated cesarean risk. Age-related variables (age and age_cat) also showed positive contributions (+ 0.0531 and + 0.0282), demonstrating the model’s sensitivity to advanced maternal age—another well-recognized clinical risk factor. In contrast, pregnancy-related complications such as HDP, APH, PROM, and risk_score exhibited small negative contributions, suggesting that these conditions played a limited role in the prediction for this particular patient. The variable regular_ANC showed no measurable effect. The congruence between the contribution patterns illustrated in Fig. 13 and the numerical values presented in Table 7 supports the internal consistency of the model’s decision process. Overall, the results indicate that the model generates individualized and interpretable predictions by systematically weighting clinically meaningful variables.

#### LIME-based consistency assessment

The stability of the SHAP explanations was further examined using LIME applied to the same clinical case. The results obtained from LIME closely paralleled the SHAP findings. Once again, mode_delivery_last emerged as the most decisive factor influencing the model’s prediction, while age-related variables showed weaker effects and the remaining predictors contributed minimally. This correspondence between the two methods indicates that the model’s decision boundaries are robust and not overly sensitive to small perturbations in the input features.

**Fig. 14:**
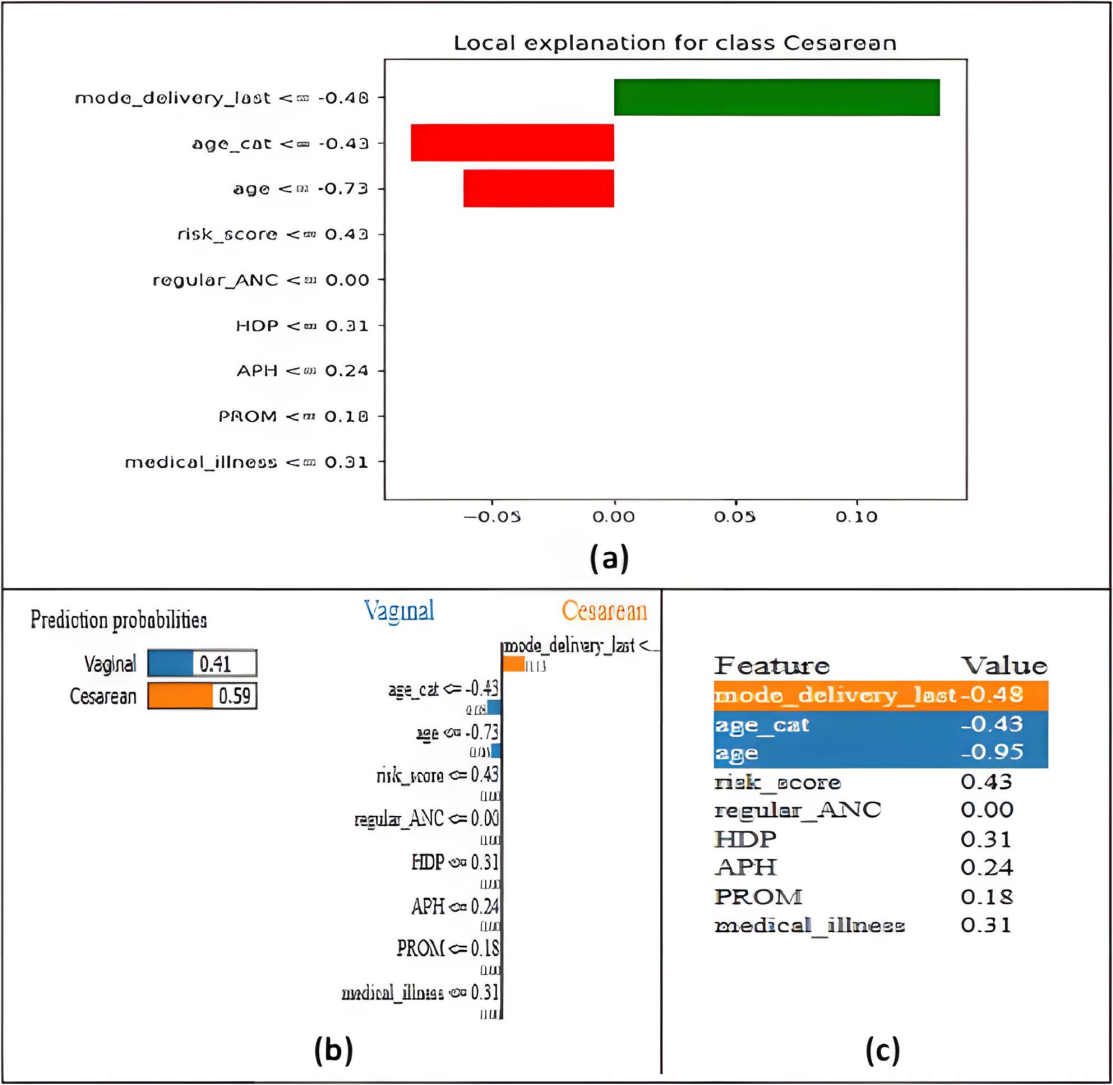
LIME-based local explanation for the selected clinical case. **a** Contribution of each feature to the cesarean prediction, **b** Predicted probabilities for vaginal and cesarean delivery, **c** Feature values used in the LIME explanation

As illustrated in Fig. 14, LIME provides a detailed decomposition of the model’s reasoning. Figure 14a highlights the local feature contributions, Fig. 14b presents the predicted probabilities for vaginal versus cesarean delivery, and Fig. 14c shows the specific feature values used in the explanation. Across all panels, mode_delivery_last maintained the strongest positive influence, reinforcing its central role in directing the prediction toward a cesarean outcome.

The features age and age_cat produced small negative contributions, suggesting that maternal age exerted a modest but noticeable effect on the decision. In contrast, risk_score, regular_ANC, HDP, APH, and PROM contributed only minimally, reflecting their limited relevance for this particular case.

The strong agreement between SHAP and LIME, in both feature rankings and contribution magnitudes, indicates that the model follows a consistent and clinically meaningful reasoning pattern. This consistency reinforces confidence in TabNet’s reliability and supports its use as a decision-support tool in obstetric risk assessment.

## Discussion and conclusions

Cesarean delivery rates continue to rise worldwide, often exceeding medical necessity. This trend has important implications for maternal and neonatal health and presents considerable challenges for obstetric management. In multiparous women, a history of previous cesarean sections combined with additional obstetric risk factors further elevates the likelihood of cesarean delivery. As a result, reliable antenatal prediction using artificial intelligence has become not only a scholarly pursuit but also a practical need to support evidence-based clinical decision-making.

This study compared classical MLP-based baseline models with advanced deep learning architectures specifically developed for tabular data in predicting cesarean delivery among multiparous women. The findings emphasize that clinical decision support requires careful consideration of predictive accuracy, explainability, generalizability, and robustness. While the MLP model served as a reasonable baseline, its enhanced version provided only limited gains. Attention-based architectures such as Attention-MLP and CBAM-MLP demonstrated performance fluctuations and signs of overfitting, likely due to the modest dataset size. In contrast, tabular-specific architectures yielded stronger and more stable performance. TabNet achieved the highest predictive accuracy (ROC-AUC = 0.79; PR-AUC = 0.74), effectively capturing key clinical patterns through its sequential attention and feature-masking mechanisms. FT-Transformer and TabPFN delivered balanced and competitive results, confirming the value of models explicitly designed for structured clinical data. Additionally, the combined use of SMOTE and weighted loss functions improved sensitivity to the minority class and strengthened clinical interpretability.

Overall, TabNet emerged as the most effective and clinically meaningful approach, providing a favorable balance of accuracy, stability, and explainability. Beyond numerical performance, the methodological contribution of this study lies in its integrated framework, which combines automated Boruta feature selection (complemented by obstetric clinical expertise) with a unified explainability pipeline built on SHAP, LIME, and TabNet’s internal feature-masking mechanism. This structured design enhances interpretability, supports clinician trust, and elevates the model beyond a direct implementation of an existing architecture. Future research should aim to validate these outcomes in diverse populations and assess integration into real-time clinical workflows to strengthen generalizability and practical utility.

### Clinical evaluation

The clinical implications of the findings are significant. Comparative analyses showed that tabular-specific deep learning models, particularly TabNet, outperform conventional MLP-based architectures in predicting cesarean delivery among multiparous women. Three dimensions are especially important from a clinical perspective: predictive accuracy, interpretability, and integration feasibility.

First, the predictive accuracy of TabNet (ROC-AUC: 0.79; PR-AUC: 0.74) is clinically meaningful. Prior research has confirmed the value of AI-driven approaches in obstetric risk prediction [3–5, 11, 32]. Our results strengthen this evidence by demonstrating that deep learning can improve antenatal risk stratification in multiparous women. Second, interpretability is central for clinical adoption. TabNet identifies key predictors such as previous cesarean delivery, hypertensive disorders of pregnancy, and antepartum hemorrhage, all of which are well established as major risk factors in obstetric practice [4, 7, 9]. This alignment with established knowledge supports physician trust and is consistent with broader recommendations emphasizing explainable AI in healthcare [6, 18, 28, 33]. Third, integration into clinical workflows represents a practical opportunity. As cesarean rates increase globally [1, 2], especially among multiparous women, embedding AI-based models into electronic health record systems could enable real-time risk prediction, improve antenatal counseling, optimize delivery planning, and reduce unnecessary procedures. Comparable integration efforts have been reported in maternal health prediction studies [10, 12, 13, 34]. Nevertheless, limitations remain. The dataset was drawn from a specific population, requiring external validation across diverse institutions. Prospective clinical trials are also needed to confirm generalizability, user acceptance, workflow integration, and patient outcomes.

In conclusion, this study demonstrates that TabNet offers a state-of-the-art, clinically interpretable, and highly accurate solution for predicting cesarean delivery in multiparous women. By integrating automated feature selection with domain-informed refinement and employing a unified explainability framework through SHAP, LIME, and TabNet’s feature-masking mechanism, the proposed approach provides a transparent and methodologically rigorous foundation for obstetric risk prediction. The model not only aligns with well-established clinical risk factors but also achieves superior performance compared with traditional and attention-based neural architectures. With further external validation and prospective implementation, this framework holds strong potential to support obstetricians in real-time decision-making, enhance antenatal counseling, reduce unnecessary surgical interventions, and ultimately contribute to safer and more effective maternal care.

### Study limitations

Several limitations should be acknowledged when interpreting the findings of this study. First, the analyses were conducted using a single, publicly available dataset comprising 460 multiparous women. Although this dataset has been previously validated and widely used in the literature, the sample size remains modest for deep learning applications. This may limit the generalizability of the proposed models; therefore, further validation on larger and independent cohorts is necessary to confirm the robustness of the results.

Second, the data were derived from a specific population and clinical setting. Variations in clinical practice, referral patterns, and regional cesarean section policies may influence model performance. Accordingly, caution is warranted when applying these findings to populations with different sociodemographic or obstetric characteristics.

A third limitation relates to the combined use of SMOTE and weighted loss functions to address class imbalance. While this strategy improved sensitivity for cesarean delivery prediction, synthetic oversampling may not fully capture the complexity and heterogeneity of real-world clinical data. Future studies should therefore explore alternative imbalance-handling approaches and evaluate model performance on prospectively collected datasets with natural class distributions.

Furthermore, although explainability was systematically assessed using SHAP and LIME, these techniques are inherently based on methodological assumptions and approximations. The absence of a standardized quantitative framework for evaluating explainability in clinical artificial intelligence limits direct comparisons across studies. Nevertheless, the consistency observed among multiple explanation methods supports the alignment of the model’s decision logic with clinically meaningful patterns.

Finally, this study is retrospective and methodological in nature. The proposed models were not evaluated in real-time clinical environments, and factors such as clinician interaction, workflow integration, and the practical impact of decision-support systems were not examined. Prospective and implementation-oriented studies are therefore required to determine the clinical utility, safety, and acceptability of explainable, model-based decision support tools in routine obstetric practice.

## Data Availability

The dataset used in this study was obtained from the supplementary materials of the study by Yimer and Mekonnen [13] entitled “Development and validation of a risk prediction model for caesarean delivery among multiparous women”. The data are publicly available within the supplementary files of the original publication. All variables used in this study and their definitions are provided in Table 1 of the manuscript.

## Abbreviations

AI: Artificial Intelligence
MLP: Multilayer Perceptron
CBAM: Convolutional Block Attention Module
ROC-AUC: Area Under the Receiver Operating Characteristic Curve
PR-AUC: Area Under the Precision–Recall Curve
SHAP: SHapley Additive exPlanations
LIME: Local Interpretable Model-agnostic Explanations
HDP: Hypertensive Disorders of Pregnancy
APH: Antepartum Hemorrhage
PROM: Premature Rupture of Membranes
